# How Do Private Providers Unaffiliated With the Nigeria National TB Program Diagnose and Treat Drug-Susceptible TB Patients? A Cross-Sectional Study

**DOI:** 10.9745/GHSP-D-22-00210

**Published:** 2022-12-21

**Authors:** Victor Abiola Adepoju, Ademola Adelekan, Victoria Etuk, Moses Onoh, Babatunde Olofinbiyi

**Affiliations:** a Department of HIV and Infectious Diseases, Jhpiego, Abuja, Nigeria.; b Blue Gate Research Institute, Ibadan, Nigeria.; c International Research Center of Excellence, Institute of Human Virology of Nigeria, Abuja, Nigeria.; d Communicable and Noncommunicable Diseases Cluster, World Health Organization, Abuja, Nigeria.; e Department of Obstetrics and Gynecology, Ekiti State University Teaching Hospital, Ado-Ekiti, Nigeria.

## Abstract

**Introduction:**

TB diagnosis and treatment practices among private providers not affiliated with the Nigeria National TB Program (NTP) have implications for TB control efforts. Studies evaluating these practices among non-NTP providers are scarce. We aimed to investigate TB diagnosis and treatment practices among non-NTP private providers in urban Lagos State, Nigeria.

**Methods:**

We conducted a cross-sectional study among doctors and nurses operating private facilities not formally affiliated with the NTP for TB case notification. Between May 2018 and January 2019, we implemented a survey using a pretested questionnaire among 152 doctors and nurses attending TB sensitization seminars in Lagos, Nigeria. We used descriptive statistics to summarize the sociodemographic information and proportion of non-NTP providers with different self-reported TB diagnostic, prescription, and monitoring practices.

**Results:**

Private non-NTP doctors and nurses self-reported diagnosing TB using 8 different types of tests. Acid-fast bacilli was the most common (39.8%) means of diagnosing TB. Private non-NTP providers also self-reported prescribing 23 different TB regimens, including streptomycin, to treat TB. Only 32.4% of providers self-reported using the correct combinations of anti-TB drugs to treat TB. Additionally, 58.3% of providers prescribed the standard 6-month treatment duration for pulmonary TB patients, and the remaining 41.7% either undertreated or overtreated TB.

**Conclusion:**

A large proportion of private doctors and nurses not formally affiliated with the NTP in Nigeria were not following the NTP guidelines in the diagnosis and treatment of TB. Overtreatment and undertreatment were common. Engagement of these practitioners by the NTP in the form of supervision, on-the-job mentorship, and other strategies can mitigate the negative effects of their current practices on TB case notification and the spread of drug-resistant strains in Nigeria.

## Abstract (precis)

Private providers unaffiliated with the National TB Program (NTP) in Nigeria are not diagnosing or treating drug-susceptible TB patients according to NTP guidelines, resulting in an urgent need to engage non-NTP providers and improve the quality of their TB services.

## Teaser

**Key Findings:**

- A majority of Nigerians seek TB care from the private sector, yet private providers who are not affiliated with the National TB Program (NTP) do not receive training on guidelines for diagnosis and treatment.
- Nearly 40% of private unaffiliated providers we surveyed self-reported using acid-fast bacilli for TB diagnosis. However, a much larger percentage used 5 other TB testing approaches not approved by the NTP to make a definitive diagnosis.
- Only 32.4% of providers surveyed prescribed the correct drug regimens. Five of the treatment regimens reported in our study contained streptomycin, which is against NTP recommendation.

**Key Implications:**

- The enormous gaps in the quality of TB diagnosis and treatment among unaffiliated NTP providers in this study have wider implications for TB control efforts, particularly case notification, spread of drug-resistant TB strains, cost of prescribing unnecessary tests, and prolonged treatment duration.
- National and subnational TB program managers need to prioritize the rapid engagement of non-NTP providers into NTP surveillance systems, routine TB update trainings, and supportive supervision and monitoring to help increase the quality of their services.

## INTRODUCTION

Nigeria is 1 of the 30 high-burden countries identified by the World Health Organization for TB, TB/HIV, and multidrug-resistant TB.^1^ The country ranks fourth in the world and first in Africa with respect to estimated number of TB cases. Nigeria also accounts for 9% of global TB notification gaps. Globally, about 10 million people developed TB in 2020 and close to 4 million of these TB cases were missed.^1^ Also, in 2020, only 138,591 of the estimated 434,000 incident TB cases in Nigeria were notified.^1^ Successful TB management involves quality diagnosis and appropriate treatment regimen, monitoring, and follow-up, failure of which may result in relapse and development of multidrug-resistant TB.^2^

There are concerns that private providers lack the knowledge to implement proper TB case management.^3^^–^^6^ A comparison of TB management in the public and private sectors in Australia noted that private-sector patients were more likely to receive less than 4 first-line medications, but treatment outcomes were comparable between sectors.^6^ A study in Nigeria to assess the quality of TB diagnosis and treatment showed that in 2008, when private sector involvement in TB control was still in its early stage, private physicians who had reported managing TB clients had poor knowledge and practice of TB management.^7^ Studies from Nigeria and India found, respec-tively, 85 and 102 different regimens were being used by private providers to treat TB.^7^^–^^8^ Similarly, a multicountry study of private TB drug markets reported about 111 different combinations of TB drugs (in both fixed and loose strengths) across 10 high-burden countries.^9^ Although 87% of private providers have managed TB in their practice in Nigeria, only 9.8% adhered to directly observed treatment, short-course (DOTS)-plus guidelines, which were available for TB care at the time of the study.^7^ Private providers in Pakistan and the Philippines agreed to use acid-fast bacilli (AFB) for TB treatment monitoring in theory, but most failed to request the test in real practice^10^^–^^11^ while cases of multiple tests to diagnose TB with enormous cost implications for patients have been reported.^8^^,^^12^^,^^13^

Private providers in Pakistan also lacked the required expertise for TB diagnosis and treatment compared with those from the public sector.^14^ In India, private providers failed to adhere to the requirements of the International Standards of Tuberculosis Care, which reechoed earlier notions that TB treatment in the unaffiliated private sector was substandard and unregulated.^15^ A study from the Philippines described how private non-NTP providers relied heavily on chest X-ray for TB diagnosis and combined inappropriate TB regimens.^12^

There are also concerns about the quality of TB care received by patients among private providers. A multicountry review showed that less than 40% of patients with TB symptoms were properly managed in private settings in studies conducted in India, Kenya, China, and South Africa, with less than 30% referred to the national TB program. Other issues related to the quality of care in the private sector include low testing and referral rates, with gaps between knowledge of TB care and correct practice of TB care by private providers.^16^

Standardized mystery clients have been used to assess TB care provided by the private sector. In India, of 250 patient-provider interactions, only 21% were appropriately managed for TB. Correct management was about 2 times higher among medical doctors.^17^ In South Africa, poor quality of TB care was reported by standardized clients, with antibiotics prescribed for TB presumptive clients.^18^ Similarly, a review of assessment of TB care using standardized patients also reported a wide variety in care and management and use of different combinations of TB drugs.^19^ Conversely, in Nigeria, results from a standardized client model study showed that private practitioners, except pharmacies, performed better than providers in public settings in managing presumptive TB cases.^20^

Although 60% of Nigerians initially sought care in the private sector when they were sick, they contributed only 11% to TB notification in 2017.^21^ Also, a previous review noted that only 56% (277/406) of private not-for-profit facilities and 5% (646/13448) of private for-profit facilities have been engaged by the National TB Program (NTP) to notify TB.^21^ However, in metropolitan Lagos, which has more than 3,500 registered private providers, less than one-third have been engaged by Lagos State TB and Buruli Ulcer Control Program (LSTBLCP) in 2019.^22^ Despite this, the contribution of private providers to TB case finding and notification in Nigeria has been increasing, with private providers contributing 31%–45% of case notifications quarterly across 3 states in Nigeria.^23^

Private non-NTP providers who are not affiliated with the NTP do not receive standardized TB/DOTS training, supervision, and tools to provide quality services that affiliated providers receive, although they may occasionally receive TB sensitization organized by the NTP. Among non-NTP providers, common shortcomings of TB case management include using GeneXpert to monitor treatment, serology for TB diagnosis, inappropriate treatment regimens, and poor case-holding skills.^24^^–^^25^ In addition, lack of awareness about the NTP notification process among non-NTP providers could also lead to underreporting.^26^

Private non-NTP providers do not receive the structured TB/DOTS training, supervision, and tools for quality service that affiliated providers receive.

A key component of the National TB Strategic Plan (2015–2020) was integrating non-NTP providers in the Global Fund Private Public Mix (PPM) program, which aimed to engage the private sector in providing more people with TB prevention, diagnosis, and treatment services. The implementation of this strategic plan would increase the country’s TB case notification by activating and integrating additional non-NTP affiliated entities (e.g., health facilities, community pharmacists, and patent medicine vendors) into the NTP surveillance and reporting systems.

There is a need for a systematic survey and needs assessment of TB case management practices specifically among non-NTP private providers. Results will help to reevaluate current PPM strategies and generate further policy discussions on engagement strategies among policy makers, program managers, service providers, and implementing partners. Our study aimed to assess how non-NTP providers diagnose and treat TB patients in Lagos State, Nigeria.

## METHODS

### Study Design

Our cross-sectional study used self-administered questionnaires to assess TB diagnostic and treatment practices among non-NTP providers across 13 local government areas (LGAs) in Lagos State, Nigeria (Supplement).

### Setting

We conducted the study in Lagos State, Nigeria, which has a population of 24.6 million people in 2015 and is divided into 20 LGAs.^27^ Each LGA is supervised by an LGA TB supervisor. Lagos has the highest TB burden in Nigeria and accounts for 11% of the national population.^20^ Health care in Lagos is organized at 3 levels: primary, secondary, and tertiary.

The Health Facility Monitoring and Accreditation Agency (HEFAMAA) is a regulatory agency embedded within the Lagos State Ministry of Health to register private and public hospitals before they can operate. The agency also supervises and monitors private and public hospitals for compliance with standards of care. More than 3,500 private hospitals in Lagos have registered with HEFAMAA in 2015. HEFAMAA uses a supervisory checklist to routinely monitor quality of care provided by all private and public hospitals in Lagos. However, the tools assess processes and practices that are not necessarily TB-specific.

In 2003, the LSTBLCP (the NTP equivalent at the subnational level) was inaugurated, and in 2008, it was expanded to engage the private sector. Engagement of private clinics with the LSTBLCP ensures TB cases detected in the private sector are notified and captured within the NTP surveillance system. Unfortunately, only less than one-third of private hospitals registered with HEFAMAA have been engaged with LSTBLCP under the PPM program while other unengaged private hospitals that may also manage TB cases fail to report to the NTP surveillance system. By 2015, Nigeria adopted GeneXpert as the first and preferred method of diagnosis for all forms of TB, and sputum AFB remains the test used for follow-up investigation.^22^

Despite attempts to engage private clinics in the NTP surveillance system that captures TB cases, many have not registered or fail to report.

In Nigeria, public and private facilities engaged by LSTBLCP manage all drug-susceptible TB patients for 6 months using a fixed-dose regimen (rifampicin, isoniazid, pyrazinamide, and ethambutol), except patients diagnosed with osteoarticular TB and TB meningitis, which are treated for 12 months. Private providers do not manage cases of drug-resistant TB (DR-TB) but instead refer them to a designated DR-TB treatment center.

We purposively selected 13 high-burden LGAs in Lagos (Alimosho, Ifako-ijaiye, Ajeromi, Ojo, Badagry, Kosofe, Apapa, Agege, Mushin, Ikeja, Oshodi, Amuwo-odofin, and Shomolu). The 13 LGAs consist of both urban and semiurban populations. We selected and recruited participants through the private-sector associations of doctors and nurses (i.e., the Association of General and Private Medical Practitioners of Nigeria and the Association of General Private Nursing Practitioners). To avoid double counting and including already engaged private providers, we requested the list of engaged private facilities from LSTBLCP and excluded any repeated non-NTP-affiliated facilities from the list shared by the private-sector associations. We leveraged quarterly association meetings to organize a TB sensitization workshop for the members. Pretested multiple choice questions were administered before the commencement of TB sensitization. The questionnaire assessed TB diagnostic and treatment practices among non-NTP-affiliated facilities. The study took place between May 2018 and January 2019.

### Participants

Participants were private sector doctors and nurses who were not formally affiliated with the NTP to provide TB services but who unofficially manage TB patients and report to the Integrated Disease Surveillance and Response systems. These include doctors and nurses from hospitals as well as nursing and maternity homes. To be included in the study, the provider needed to own, manage, or comanage a private practice (private hospital, nursing, or maternity home), not be engaged by the NTP, be aged 18 years or older, and have diagnosed at least 1 TB case in the past. We excluded private providers who were already trained and engaged by the NTP to notify TB, who had never diagnosed any TB case in the past, or who were younger than 18 years. Providers from both urban and semi-urban areas and from private hospitals of various sizes were included to avoid selection bias.

### Sampling Technique

A 4-stage sampling technique (summarized below) was used to select participants for the study. Sample size was determined using the formula

$${n=a}^{2}{b/d}^{2}$$

where n=sample size, a=Z statistic for a level of confidence, b=prevalence, and d=precision or confidence interval.

The level of a=1.96 and d=0.05 at 95% confidence interval. Dosumu et al. previously estimated 9.8% as the prevalence of knowledge and practice of TB DOTS-plus among private providers in Nigeria, resulting in b=0.098.^7^ With a nonrespondent rate of 5%, the final sample size of 152 was used in the study.

#### Stage 1: Selection of Study LGAs

We purposively selected 13 LGAs with a high TB burden and private sector presence in Lagos, Nigeria.

#### Stage 2: Selection of Private Providers

We stratified the TB facilities in Lagos into private NTP-affiliated and private non-NTP-affiliated categories and used a convenience sampling technique to select the private non-NTP-affiliated facilities from the target LGAs.

#### Stage 3: Selection of Private Non-NTP Health Facilities

We filtered all the NTP-affiliated private facilities from the universal list of all private facilities by cross-checking the NTP list with those of HEFAMMA and private sector associations. We used a systematic random sampling approach to select the required numbers of assessment private non-NTP facilities in each of the LGAs in a representative sample of LGAs in the state, using Research Randomizer (www.randomizer.org) to produce random numbers from the list of private non-NTP affiliated facilities in each of the 13 LGAs in Lagos state.

#### Stage 4: Selection of Non-NTP Providers From the Non-NTP-Affiliated Private Facilities

We used a purposive sampling technique to select the required numbers of health care providers (doctors and nurses) interviewed in the selected health facilities. We selected the medical director (a doctor) for the private medical facility and the nursing director for private nursing homes. Where the medical director or nursing director was not available in a facility, we selected a representative doctor or nurse who met the inclusion criteria. If more than 1 health worker met the inclusion criteria, we used simple random balloting to select respondents to be interviewed.

### Variables

We included both categorical and continuous variables. Categorical variables included age group, gender, type of health facility, practice setting, outpatient department attendance, non-NTP qualification, previously managed TB patient, and previous TB training. Continuous variables included years of practice and number of years since last TB training. Continuous variables were recoded into categorical based on median value. For yes or no questions, yes was coded as 1 and no as 0. Open-ended responses were categorized based on the type of responses and coded accordingly.

### Data Sources and Measurement

The questionnaires included 17 questions in 2 parts. Part 1 comprised 13 closed-ended questions on participants’ sociodemographic characteristics, such as age, gender, outpatient department attendance, practice setting, qualification, and years of experience in TB diagnosis and treatment.

Part 2 comprised the following 4 open-ended questions to assess TB diagnostic practice, treatment monitoring practice, treatment duration, and TB regimen prescription practice.

- Mention the combination of anti-TB drugs used to treat drug-susceptible pulmonary tuberculosis in your hospital.
- For how many months do you treat drug-susceptible pulmonary tuberculosis in your hospital?
- What diagnostic test do you use to diagnose TB in your hospital?
- What test do you use to follow up drug-susceptible TB patients on treatment in your hospital?

We pretested the questionnaires across 10 selected non-NTP facilities that were not part of the study. The LSTBLCP reviewed the questionnaires and gave input. Subsequently, participants self-administered the questionnaire following extensive explanation and preinstructions by the study coordinator in a bid to minimize completion errors. Participants received additional clarification about the questions where needed. Once participants completed the questionnaires, they were double-checked for completeness and any omissions were addressed unless respondents declined. The questionnaire completion took approximately 60–70 minutes on average. Responses to the open-ended TB diagnostic and treatment practices were evaluated against recommendations in the NTP guidelines to determine correct and incorrect responses. Different open-ended responses were coded, analyzed, and presented as frequency and proportions. Non-NTP providers were subsequently sensitized after the test, followed by revision of the test.

### Data Collection and Analysis

Trained independent data entry clerks first entered the data into Microsoft Excel. Data collected were then categorized, coded, and cleaned before being imported into SPSS version 22 for additional statistical analyses. In addressing the research objectives, we applied descriptive statistics to summarize the sociodemographic status and described the proportion of participants with different TB diagnostic and treatment practice responses. Incorrect and correct practices were presented using frequency and percentages based on provision of the NTP guidelines.

### Ethical Approval

Ethical approval was obtained from the Ethical Committee of the Lagos State University Teaching Hospital. Permission was also received from the LSTBLCP. All study participants gave their informed consent. All information from participants was kept anonymous in conformity with the ethical guidelines.

## RESULTS

### Participants

The majority of participants were private for-profit providers (139 [91.4%]); women (89 [58.6%]); general practitioners (58 [38.2%]); and aged 20–34 years (56 [36.8%]) (Table 1). Fifty-seven providers (37.5%) consulted an average of <100 general patients monthly and 73 providers (48%) had spent 1–16 years in practice.

**TABLE 1.** Demographic Characteristics of Non-NTP Providers, Lagos, Nigeria

**Provider Characteristics**	**Providers, No. (%)** **(N=152)**=
Age, years	
20–34	56 (36.8)
35–49	46 (30.3)
50–64	40 (26.3)
>65	10 (6.6)
Gender	
Female	89 (58.6)
Male	63 (41.4)
Type of health facility	
Private not-for-profit	13 (8.6)
Private for-profit	139 (91.4)
Monthly outpatient department patients treated	
<100	57 (37.5)
100–200	46 (30.3)
200–500	33 (21.7)
>500	16 (10.5)
Qualification	
MBBS/General practitioner	60 (39.5)
Nurse/Midwife	92 (60.5)
Time in clinical practice, years	
1–16	73 (53.3)
17–44	64 (46.7)
Previously managed TB patient	
No	61 (40.1)
Yes	91 (59.9)
Prior participation in TB-specific training	
No	106 (69.7)
Yes	46 (30.3)
Time since last TB training, years	
<1	9 (19.6)
1–5	18 (39.1)
5–10	14 (30.4)
>10	5 (10.9)

Abbreviations: MBBS, Bachelor of Medicine and Bachelor of Surgery; NTP, National TB Program.

Overall, non-NTP providers reported prescribing 23 different anti-TB combination regimens (Table 2). Of these 23 regimens, 5 contained streptomycin. Of the total 108 non-NTP providers that responded to the question on prescribed regimen for the treatment of drug-susceptible pulmonary TB (PTB), only 35 (32.4%) used the correct regimen (rifampicin, isoniazid, pyrazinamide, and ethambutol) while the remaining 67.6% self-reported using different incorrect combinations of anti-TB drugs.

**TABLE 2.** Prescription Practices for Anti-TB Drug Regimen Among Non-NTP Providers, Lagos, Nigeria

**Anti-TB Drug Regimen**	**Providers, No. (%)** **(N=108)**
Regimen recommended by NTP	
Rifampicin, isoniazid, pyrazinamide, and ethambutol	35 (32.4)
Regimen(s) not recommended by NTP	
Isoniazid and rifampicin	11 (10.2)
Ethambutol, isoniazid, and rifampicin	10 (9.3)
Isoniazid, rifampicin, and streptomycin	10 (9.3)
Isoniazid	6 (5.6)
Ethambutol, isoniazid, and pyrazinamide	4 (3.7)
Isoniazid, pyrazinamide, rifampicin, and streptomycin	4 (3.7)
Rifampicin, isoniazid, pyrazinamide, ethambutol, and pyridoxine	4 (3.7)
Rifampicin and streptomycin	4 (3.7)
Isoniazid, pyrazinamide, and rifampicin	3 (2.8)
Ethambutol, pyrazinamide, and rifampicin	2 (1.9)
Ethambutol and rifampicin	2 (1.9)
Isoniazid and pyridoxine	2 (1.9)
Streptomycin injection	2 (1.9)
Cefritriaxone, gentamycin, rifampicin, and streptomycin	1 (0.9)
Cotrmoxazole and ethambutol	1 (0.9)
Ethambutol and isoniazid	1 (0.9)
Ethambutol, isoniazid, pyridoxine, and rifampicin	1 (0.9)
Ethambutol, isoniazid, rifampicin, and streptomycin	1 (0.9)
Ethambutol, isoniazid, rifampicin, and thiacetazone	1 (0.9)
Ethambutol, pyrazinamide, and streptomycin	1 (0.9)
Isoniazid, pyridoxine, and rifampicin	1 (0.9)
Rifampicin, isoniazid, pyrazinamide, and pyridoxine	1 (0.9)
Total	108 (100)

Abbreviation: NTP, National TB Program.

The majority (63 [58.3%]) of the 108 respondents reported treating TB patients for 6 months per the NTP guidelines (Table 3). However, 24 providers (22.3%) reported undertreating for less than 6 months, and 21 providers (9.4%) reported overtreating PTB patients for more than 6 months.

**TABLE 3.** Duration of TB Treatment Ordered by Non-NTP Providers, Lagos, Nigeria

**Duration of TB Treatment, Months**	**Providers, No. (%)**
6	63 (58.3)
≤3	18 (16.7)
3–6	6 (5.6)
6–12	21 (19.4)
Total	108 (100)

Abbreviation: NTP, National TB Program.

Non-NTP providers in Lagos used 8 different tests to diagnose TB: AFB; chest X-ray; sputum microscopy, culture, and sensitivity; GeneXpert; Mantoux; full blood count; erythrocyte sedimentation rate; and QuantiFERON-TB (Table 4). A high number of participants (43 [39.8%]) used AFB to diagnose TB, followed by chest X-ray (29 [26.9%]) and sputum microscopy, culture, and sensitivity (14 [13%]). Only 8 providers (7.4%) used GeneXpert for diagnosis.

**TABLE 4.** Type of TB Diagnostic Test Ordered by Non-NTP Providers, Lagos, Nigeria

**Type of TB Test**	**Providers, No. (%)**
Tests recommended by NTP	
Acid-fast bacilli	43 (39.8)
Chest X-ray	29 (26.9)
GeneXpert	8 (7.4)
Tests not recommended by NTP	
Sputum MCS	14 (13.0)
Mantoux	12 (11.1)
FBC, ESR	1 (0.9)
QuantiFERON-TB	1 (0.9)

Abbreviations: ESR, erythrocyte sedimentation rate; FBC, full blood count; MCS, microscopy, culture, and sensitivity; NTP, National TB Program.

## DISCUSSION

Our objective was to highlight TB diagnostic and treatment practices among non-NTP providers in Lagos, Nigeria. Our findings show that non-NTP providers reported diagnosing TB using 8 different diagnostic tests. The most common method was AFB used by 39.8% of respondents. A study from rural Pakistan also reported the use of 3 different tests (erythrocyte sedimentation rate, Mantoux, and chest X-ray) by non-NTP affiliated facilities to diagnose TB. The study added that non-NTP providers relied on clinical judgment and chest X-rays for TB follow-up.^24^ Our finding raises concern about the use of inappropriate TB diagnostic tests not approved per the NTP guidelines. Relying on substandard serologic diagnostic tests like QuantiFERON-TB gives faster and immediate results but lacks specificity for active TB, which can lead to overtreating patients who might not have had active TB. This finding is further echoed by a review of 22 TB high-incident countries that highlighted the global spread of the use of serological tests for TB diagnosis.^25^ The researchers called for immediate scale-up and optimization of NTP-approved diagnostic tests (e.g., GeneXpert, culture, microscopy, chest X-ray) and adoption of new tools to aid faster TB diagnosis, which is crucial for private providers. Private providers need to be better trained and kept abreast of appropriate diagnostic tests and their benefits per the NTP guidelines. More importantly, private providers need support for quality sputum assessment and transportation for GeneXpert diagnosis with the quickest possible turnaround.

Non-NTP providers relied on clinical judgment and chest X-rays for TB follow-up, which raises concern about the use of inappropriate TB diagnostic tests not approved per the NTP guidelines.

Non-NTP providers in Lagos reported treating TB using 23 different drug combinations. Studies from Nigeria and India found much higher numbers of drug combinations, 45–85 and 102, respectively, that were inappropriately prescribed as TB treatment regimens.^7^^–^^8^ However, our finding is higher than reports from Kenya and Argentina, which found that private practitioners in each of the studies used 16 different combinations of first-line anti-TB regimens for treatment.^4^^,^^28^ Five of the regimens reported in our study contained streptomycin, which is against the NTP recommendation. Streptomycin has the highest resistance of 27.6% among first-line anti-TB drugs and has been excluded from the list of first-line drugs in Nigeria.^29^ Our finding of its continuous use among private providers has serious implications for the spread of DR-TB and for the prevalence of its side effects among pregnant women.

Five of the regimens reported in our study contained streptomycin, which has been excluded from the list of first-line anti-TB drugs in Nigeria due to its high resistance.

Only 32.4% of respondents in this study reported prescribing appropriate first-line TB regimens per the NTP guidelines. This is lower than the 60% of private practitioners found in the Philippines^11^ but higher than findings of 27.3%, 29.4%, and 11% among private practitioners respectively from Indonesia, India, and Korea.^8^^,^^30^^,^^31^ Studies from India found that 12%–45.5% prescribed second-line drugs for new adult PTB patients while another study from West Bengal, India noted that 27% of practitioners gave alternate day regimens.^4^^,^^32^ The exact reasons for the prescription of nonfixed-dose combinations were not clear. However, the majority of these studies were conducted in the early days of DOTS expansion into the private sector, and non-NTP providers only had access to loose tablets since fixed-dose combinations were only available in private clinics formally affiliated with NTPs. Poor TB case management, as we found in this study, increases the risk of DR-TB, which is of great concern among non-NTP providers.

We found that only 58.3% of private non-NTP providers reported placing PTB patients on a standardized 6-month anti-TB regimen as recommended by the NTP, while the remaining 41.7% self-reported overtreating (>6 months) or undertreating (<6 months) TB patients. Private non-NTP providers from India also failed to manage TB for the appropriate 6-month recommended duration.^33^^–^^34^ In our study, 22.3% and 19.4% of non-NTP providers undertreated and overtreated TB respectively. This finding is higher than 18% and 15% of private practitioners that respectively undertreated and overtreated TB in India,^33^ and 9.4% and 11.7% that respectively undertreated and overtreated TB in another Nigeria study,^7^ although the sample size in the Nigeria study was small. However, this finding is lower than results from 2 different studies in India that reported overtreatment of 51.3% and 49%, respectively.^8^^,^^34^ Overtreatment reduces adherence to medications and increases the chances of drug toxicity and side effects as well as the catastrophic cost of TB treatment. Undertreatment also increases the chances of DR-TB strains. Alternate day or irregular treatment could also give rise to resistance to first-line anti-TB drugs.^35^

### Recommendations

Considering the public health impact of poor-quality diagnostic and treatment services among non-NTP providers, policy makers and program managers should consider designing innovative approaches and rapidly scaling up engagement of these providers, strengthening their capacity, and monitoring their adherence to TB management guidelines. Urgent attention is needed to improve the quality and reach of the PPM program among non-NTP providers. On-the-job mentorship, direct observation of practices, and regular supervision could improve their TB diagnostic and treatment practices. These interventions should take into consideration the busy schedule of non-NTP providers and their desire for incentivization. In addressing the quality of TB care provided by non-NTP providers, innovations aimed at regulation such as linking annual facility accreditation with quality of care should be deployed. Future research should complement the type of survey we conducted with qualitative research to gain deeper insight into the reasons for the TB diagnostic and treatment practices among private non-NTP providers.

On-the-job mentorship, direct observation of practices, and regular supervision could improve the TB diagnostic and treatment practices of non-NTP providers.

### Limitations

This study is cross-sectional and relied on self-reported TB diagnostic and treatment practices of non-NTP providers rather than actual practice through direct observation or simulation. It is not uncommon to have discrepancies when self-reported practices are subjected to methodologies that seek to unravel actual provider practices. Furthermore, the risk of social desirability bias could lead to overreporting of TB case management practices. However, the study has contributed to the body of knowledge on diagnostic and treatment practices among non-NTP providers in Nigeria, documenting recent evidence of minimal progress and making recommendations to address quality issues. The foundation and evidence generated will provide strategic guidance while engaging non-NTP providers in Nigeria and other settings under the National PPM Roadmap.

## CONCLUSION

Our study revealed that a large number of non-NTP providers in Nigeria did not follow NTP recommendations in their TB diagnosis and treatment. Many providers used non-NTP-approved TB investigations to diagnose TB and prescribed inappropriate TB regimens. Non-NTP providers also subjected TB patients to either overtreatment or undertreatment. These findings have negative implications for TB case notification and the spread of DR-TB strains in Nigeria. Since private-sector providers will continue to attract a large number of patients in urban settings, their successful engagement by the NTP is critical to addressing the TB notification gap and the menace of DR-TB in Nigeria.

## Supplementary Material

22-00210-Adepoju-Supplement.pdf

## Notes

**Peer Reviewed**

**First published online:** November 18, 2022.
