# Supplementary material for: How Do Private Providers Unaffiliated With the Nigeria National TB Program Diagnose and Treat Drug-Susceptible TB Patients? A Cross-Sectional Study
Source: Glob Health Sci Pract. 2022 Dec 21;10(6):e2200210. doi: 10.9745/GHSP-D-22-00210 (PMC9771464; doi:10.9745/GHSP-D-22-00210)
Supplement: 22-00210-Adepoju-Supplement.pdf [file 22-00210-Adepoju-Supplement.pdf]

Supplement to: Adepoju VA, Adelekan A, Etuk V, Onoh M, Olofinbiyi B. How do private providers unaffiliated with the Nigeria National TB Program diagnose and treat drug-susceptible TB patients? A cross-sectional study. *Glob Health Sci Pract*. 2022;10(6). https://doi.org/10.9745/GHSP-D-22-00210

Supplement. Questionnaire to assess tuberculosis management practices of unengaged private doctors and nurses, Lagos, Nigeria

SECTION ONE: DEMOGRAPHIC CHARATERISTICS OF PRIVATE PROVIDERS

1. Age in years a. 18-34 years b. 35-49 years c. 50-64 years d.> 65 years
2. My facility is a. Private for-profit b. Private not for profit
3. Gender a. Male b. Female
4. Average monthly OPD attendance a. <100 b. 100-200 patients c.201-500 d >500 patients
5. Qualifications a. MBBS/GP b. Nurse c. Midwives d. Nurse/Midwives e. Auxiliary Nurse f. CHEW g. specialist doctor
6. How many years Post graduation (please write the number of years)....................
7. How many years of practice (please write the years of practice experience)..................
8. Are you aware of the National and Lagos State TB and Leprosy and Buruli Ulcer Control Program? A. Yes b. No
9. Have you managed TB patients before a. Yes b. No
10. On the average how many TB patients have you managed before.....................
11. Have you participated in any TB-specific training before a. Yes b No
12. If yes, when last did you participate in the TB training............ a. < 1 years b. 1-5 years c. 5-10 years d. > 10 years
13. Have you heard about DOTS before a)Yes b)No

SECTION 2: PROVIDER PRACTICES OF TB DIAGNOSIS, MONITORING AND TREATMENT

1) Mention the combination of anti-TB regimen you use to treat drug-susceptible Pulmonary Tuberculosis (PTB) in your hospital...

2)For how many months do you treat drug-susceptible PTB patients in your hospital........................

3)What diagnostic test do you use to diagnose TB in your hospital (List all)........................

4)What test do you use to follow up Drug susceptible TB patients on treatment in your hospital........................?
